# Lactose-riched Mongolian mare's milk improves physical fatigue and exercise performance in mice

**DOI:** 10.7150/ijms.53098

**Published:** 2021-01-01

**Authors:** Yi-Ju Hsu, Wei-Lun Jhang, Mon-Chien Lee, Batsuren Bat-Otgon, Erdenechuluun Narantungalag, Chi-Chang Huang

**Affiliations:** 1 Graduate Institute of Sports Science, National Taiwan Sport University, Taoyuan City 33301, Taiwan.; 2 School of Physical Education, Mongolian National University of Education, Ulaanbaatar, Mongolia.

**Keywords:** mare's milk powder, anti-fatigue, lactate, glycogen storage

## Abstract

Fatigue may cause the efficiency of the organ in human body to decrease, which may affect the daily life and exercise performance of the general people and athletes. Mare's milk powder (MMP) is a lactose rich supplement. The research of the study is to evaluate the whether MMP has anti-fatigue effect. Forty male ICR mice were randomly divided into four group to receive vehicle or MMP by oral gavage at 0 (Vehicle), 0.27 (MMP-1X), 0.54 (MMP-2X), 1.35 (MMP-5X) g/kg/day for 14 days. The forelimb grip of the MMP-2X, and MMP-5X group were significantly higher than the vehicle group. The swim-to-exhaustion times of the MMP-1X, MMP-2X, and MMP-5X group were significantly greater than the vehicle group. Glycogen levels in liver and muscle were significantly larger in the MMP-1X, MMP-2X, and MMP-5X groups than the vehicle group. Receive MMP supplement for 14 days can promoting exercise performance and amelioration of exercise-induced fatigue.

## Introduction

Mare's milk is popular in areas of Mongolia, China, Central Asia, Russia and Kazakhstan 1. Previous research has reported that the mare's milk is usually used for diet therapy and can be used for complementary purposes in tuberculosis, chronic hepatitis B, gastric ulcer, psoriasis, intestinal inflammation, anemia and postoperative recovery since mare's milk consists of a high concentration of polyunsaturated fatty acid 2, 3. Traditionally, fresh mare' milk is naturally fermented to produce Mongolian mare's milk, which a long history of consumption in the Mongolian steppes 4, 5. Mare's milk products play an important role in Mongolia's food culture, which contains a similar amount of crude protein, salt, lactoferrin and lactose but a lower amount of fat with a greater proportional of poly-unsaturated fatty acid when compared with the human milk 6. Previous study has shown that hydrolysates of mare's milk proteins, contains vitamins A, E, and C, an essential nutrient which have antioxidant activity, improvement in exhausted swimming time, decrease levels of lactate and urine in the blood, effects on degradation product in the process of lipid peroxidation and antioxidant enzyme levels 7. Milk and dairy products are a very good source of protein, amino acids, vitamins and minerals. A growing number of studies discuss the benefits of milk intake on exercise recovery, exercise performance and muscle function recovery, but the overall results were inconsistent 8. Mare's milk shows high biological activity ingredients, which health-promoting benefit the human body 9.

In recent years, a growing number of athletes have recognized post nutrition as optimization of exercise performance. A variety of evidence-based marketing products spring up with supporting ergogenic value, including energy repletion, protein, vitamin, mineral and phytochemical supplementation with the function of health promotion, strength improvement, muscle and weight gain 10. An investigation in 2007 reported that most athletes (including about 85% elite track and field athletes) took dietary supplements in their regular training and competition session 11. There is a growing tendency of using sport nutrition supplements for performance, which represents the supplements become an important part for athletes 12. Ingestion of carbohydrate is known to promote competition performance, it is also produces several beneficial effects, such as delay or reduction of central fatigue, delay the depletion of muscle glycogen (e.g. muscle glycogen sparing), a reduction of stress hormones and inflammatory cytokines, and support high daily training volumes and intensities and promote optimal recovery 13, 14. The mare's milk powdered is rich in high lactose. Lactose is a disaccharide that composed of glucose and galactose, which is the main source of energy of infant mammals 15. In a prior study of trained male cyclists, the consumption of galactose 30 minutes before a cycling endurance test could maintain and higher blood glucose levels throughout exercise for exercising muscles metabolism, as well as longer to exhaustion 16.

Fatigue caused by exercise is a very common sensation, which everybody has experienced. Two of the main theories of exercise regulation are the muscles themselves (peripheral mechanisms) and reside in the central nervous system (central governor) model of fatigue 17. Muscle fatigue is a commonly experienced phenomenon that limits athletic performance and other strenuous or prolonged activity, is mainly manifested as decreased exercise capability, several possible mechanisms, such as nervous, ion, vascular and fuel systems, metabolic factors (cortisol, catecholamine, interleukin-6) and fatigue reactants (hydrogen ions, lactate, inorganic phosphate, reactive oxygen species) 18, 19. Theory suggests that during exercise, many energy sources, such as glucose and liver glycogen, will be exhausted, thus leading to physical fatigue 19. Nutrient supplementation could positively enhance exercise capacity and delay the onset of fatigue, researches attempt to seek anti-fatigue natural nutritional supplement to accelerate eliminating fatigue 20, 21. The main performances of fatigue are reductions on the maximum output power, induced inability to perform the expected work output during exercise 22. Physical fatigue can be accompanied by deterioration in functional performance 21. However, still relatively no study has been reported on anti-fatigue function effect of mare's milk. Therefore, the objective of this research was to evaluate the anti-fatigue activity of mare's milk using a mice model.

## Materials and methods

### Animals and Experiment Design

The mare's milk powder (MMP) was obtained from Mongolian National University of Education, School of Physical Education. The content of nutrients and lactose in mare's milk powder were analyzed by SGS Taiwan, Ltd. (New Taipei City, Taiwan) (**Table 1**). Forty male Institute of Cancer Research (ICR) mice aged 8 weeks were obtained from the specific pathogen free (SPF) animal laboratory of BioLASCO, Yi-Lan, Taiwan. All mice were housed at the animal facility of Graduate Instituted of Sport Science at National Taiwan Sport University. The animal rooms are maintained at 22±2°C with a relative humidity of 60%±10%. Conditions under a 12-hour light-dark cycle were maintained with automatic timers. During the experiment, mice were fed by distilled water and standard laboratory chow diet (No. 5001; PMI Nutrition International, Brentwood, MO, USA) ad libitum. All experiments involving the mice and all the care and handling of the mice were performed using protocols approved by the Institutional Animal Care and Use Committee (IACUC) of National Taiwan Sport University (No. 10715). All methods were performed under the relevant guidelines.

**Figure 1:**
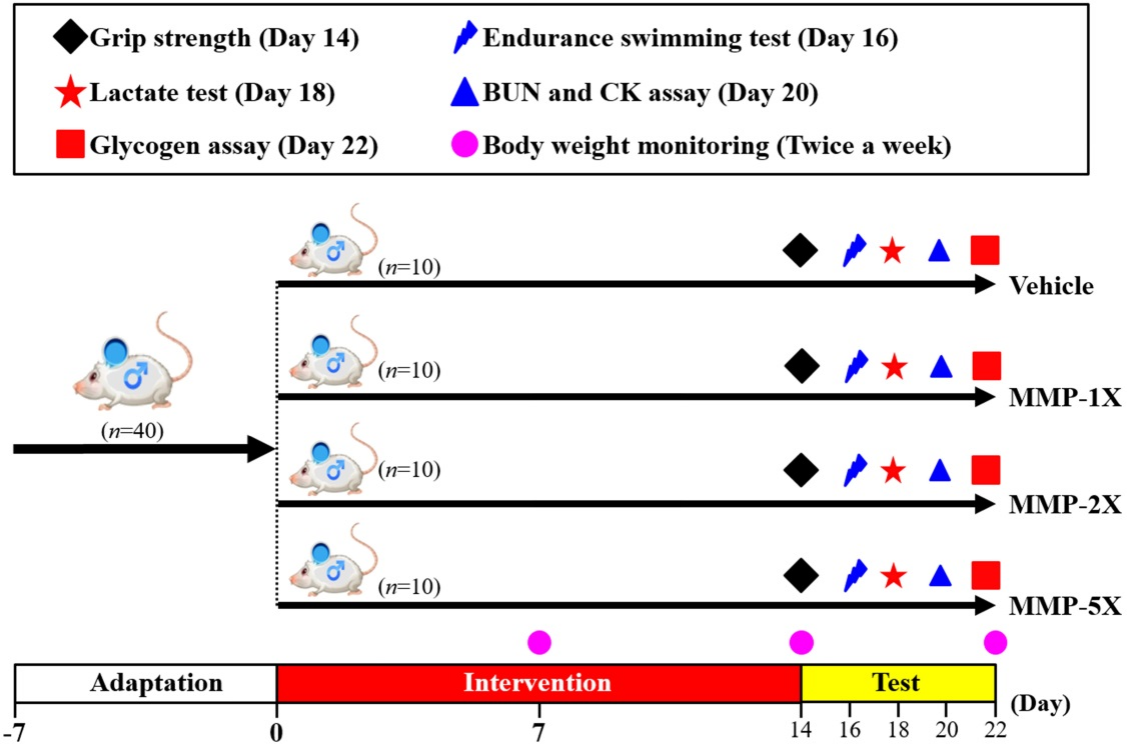
Experimental designs for the effects of MMP on exercise adaptation. The animals were randomly assigned to the four groups indicated (vehicle, MMP-1X, MMP -2X, and MMP -5X) and were consecutively supplemented with MMP until the end of the experiments. The physical capacities and related biochemistries were assessed within the test duration.

The detailed experimental procedure is illustrated in **Figure 1.** Acclimated to our vivarium for one week, and then randomly assigned to one of four groups (n = 10 mice/group) for oral gavage treatment with mare's milk once daily for 14 consecutive days: (1) vehicle control; (2) 270.6 mg/kg MMP (MMP-1X); (3) 541.2 mg/kg MMP (MMP-2X); and (4) 1353 mg/kg MMP (MMP-5X). Vehicle control group is treated with the same solution of distilled water to body weight (BW). For each mouse, average daily food and water intakes were monitored daily throughout the experiment, and recording the weight weekly.

### Mare's milk supplementation

The daily dose of MMP is 1300 mg/day for humans. On the basis of weight/surface area, the conversion between human adult dose and mouse dose is a coefficient of 12.3. For the 1X MMP dose, the mouse dose used was 1300 (mg)/60 (kg) = 21.67, 21.67 × 12.3 (the conversion factor) = 270.6 mg/kg mouse dose.

### Grip strength test

On the 14^th^ day, use of low force testing system Model-RX-5, Aikoh Engineering, Nagoya, Japan was used to measure forelimb of mice after 30 minutes feeding. The tensile force was measured using a force transducer equipped with a 2 mm in diameter and 7.5 cm long metal bar for each mouse. The mice were allowed to grip the pull bar on the grip wire with only their front paws was steadily pulled back until they lost their grip with the metal bar. The detailed procedures have been described in our previous reports 23. Grip strength was measured 10 times and the peak tension during each trial was recorded with the attached force gauge. The maximal force (in grams) recorded using this low-force system was used as the grip strength.

### Exhaustive swimming exercise

Endurance performance is an important parameter for evaluating anti-fatigue, evaluated by an exhaustive swimming test. On the 16th day of the experiment, 30 minutes post MMP administration, each animal was submitted to exhaustive swimming test with intensity equivalent to overload of 5% of the body weight tagged to the tail, then was evaluated in a columnar swimming pool maintained at 28±1°C, the swimming pool is 65 cm high, 40 cm diameter, 40 cm deep. Each mouse was loaded with a lead block, weighting approximately 5% of the body weight tagged to the tail. The endurance performance of each mouse was measured as the swimming time, recorded from the beginning to exhaustion. The mice were determined to be exhausted when uncoordinated movements occurred with failure to swim to the surface within a 7-second period 24. The exhaustive swimming time was used as an index of exercise endurance.

### Acute 10 minutes Swimming Test

The serum lactate levels were evaluated after 2 weeks of MMP treatment, 30 minutes after the final oral administration, the mice were forced to swim for 10 minutes without any weight loading. The fatigue-related variables were assessed under fasting conditions to reflect the real physiological adaptation during the acute exercise challenge. Under fasting conditions, we examined blood lactate concentrations in mice before exercise, after 10 min swimming exercise, and 20 min rest during the acute exercise challenge. Serum samples were obtained by centrifugation of blood samples at 1000× g for 15 minutes at 4 °C and were analyzed by an autoanalyzer (Hitachi 7060, Hitachi, Tokyo, Japan). Besides, the lactate production rate was calculated as the post-exercise rate divided by the before exercise rate (B/A), and the lactate difference between the post-exercise rate and the post-rest rate divided by the post-rest rate was defined as the clearance rate.

### The 90 minutes free swimming test

On the 20^th^ day of the experiment, after the treatment with MMP or distilled water, the mice were allowed to rest for 30 minutes. The mice were forced to swim for 90 minutes without weight loads. At the end of the swim, the mice rested for 60 minutes before blood samples were taken for analysis of creatine kinase (CK) and urea nitrogen (BUN). Serum samples were obtained by centrifugation of blood samples at 1000× g for 15 minutes at 4 °C and were analyzed by an autoanalyzer (Hitachi 7060, Hitachi, Tokyo, Japan).

### Tissue sample preparation

All animals were euthanized with 95% CO_2_ asphyxiation 48 h after the last treatment, and blood was immediately collected. After the mice were euthanized, the pancreas, epididymal fat pad, perirenal fat pad, and retroperitoneal fat pad were excised and weighed. Organs and tissues were excised, rinsed in saline solution and blotted dry. The whole weight and the specific tissue weight (%) relative to the individual body weight were recorded and calculated.

### Blood biochemical assessments

The pancreas, liver, kidney, epididymal fat pad and brown adipose tissue (BAT) were removed and fixed in 10% formalin for 24 hours. Tissues were embedded in paraffin and cut into 4 μm thick slices for morphological and pathological evaluation. Sections were stained with hematoxylin-eosin (H&E) and examined under a microscope equipped with a charged couple device (CCD) camera (BX-51, Olympus, Tokyo, Japan).

### Glycogen content analysis

The stored form of glucose is glycogen, which exists mostly in liver and muscle tissue. Liver and muscle tissues were excised after the mice were sacrificed and weighed for glycogen content analysis as we described previously 25. Parts of the liver and muscle tissues were stored in liquid nitrogen for glycogen concentration analysis, as described previously 25. Briefly, 100 mg of liver and muscle tissue was finely cut, weighed, and homogenized in 0.5 cold perchloric acid. After centrifugation for 15 minutes at 15,000× g and 4 °C, the supernatant was discarded. Standard glycogen (Sigma, USA) or tissue extracts (30 µL) were added to wells of a 96-well plate, followed by an iodine-potassium iodide reagent (200 µL). The plate was allowed to rest for 10 min before the absorbance was measured at 460 nm by an ELISA reader.

### Histological staining of tissues

Pancreas tissue sections (4 μm) were stained with haematoxylin-eosin. The sections were placed in a Bond Max automated stainer (Leica Biosystems, Australia) according to the following protocol. First, samples were deparaffinized and pre-treated with the epitope retrieval solution (EDTA-buffer) at 100 °C for 20 minutes. Then they were washed and peroxidase blocking was carried out for 5 minutes using the Bond Polymer Refine Detection Kit DS9800 (Leica Biosystems, UK). Tissues were washed and then incubated with the primary antibody Glucagon (clone EP74, Zeta Corporation, USA) and Insulin (clone EP125, Zeta Corporation, USA) for 45 minutes. After being washed, samples were incubated with secondary antibody (DS9800, Leica) for 15 minutes before being developed with 3,3'-diaminobenzidine tetrahydrochloride (DAB) for 5 minutes. Finally, slides were counterstained with hematoxylin for 7 minutes.

### Statistical analysis

Data are presented as means±standard error mean (SEM). Data were analysed by one-way analysis of variance (ANOVA) with SPSS for Windows, version 24.0 (SPSS Inc., Chicago, IL, USA). Tukey's test was used to measure the difference between different treatments. The Cochran-Armitage trend test was used to assess dose-responsiveness) with SAS version 9.0 (SAS Inst., Cary, NC). Differences were considered significant at *p* < 0.05.

## Results

### Effects of MMP supplementation on food and water consumption, body weight, and organ weight

**Figure 2:**
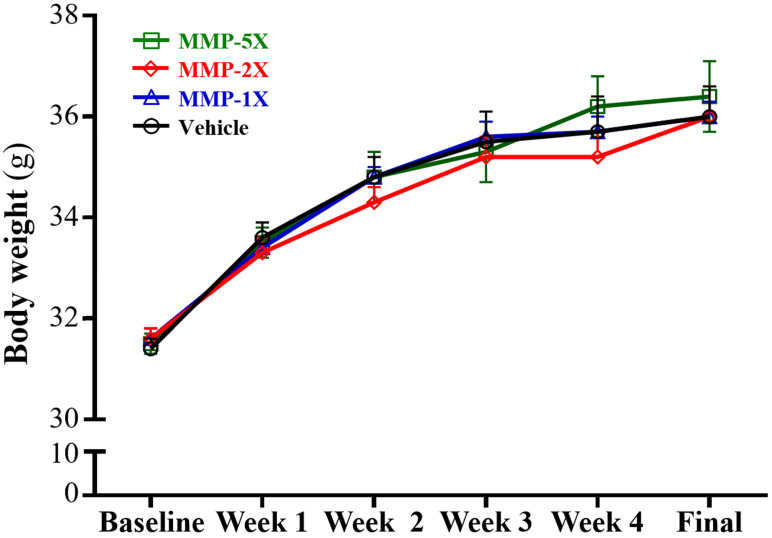
Effect of MMP supplementation on body weight. Data are mean±SEM for n =10 mice per group.

After the experiment began to intervene, the body weight sustained steady increase and no significantly between each group until to the end (**Figure 2**). In **Table 2**, there were no significant differences in food and water intake among each group.

**Table 2** shows that body compositions, the EFP tissue weight was significantly lower in MMP-1X, MMP-2X and MMP-5X than vehicle group, by 25.9% (*p* =0.013), 22.8% (*p* =0.034) and 26.6% (*p* =0.010) respectively. The relative muscle EFP was lower in the MMP-1X, MMP-2X and MMP-5X than vehicle group 25.65% (*p* =0.022), 22.39% (*p* =0.047) and 26.73% (*p* =0.015), respectively. In the trend analysis, the relative EFP weight (%) dose-dependently increased as the MMP dose (*p* =0.0006, *p* <0.0001) decreased.

### Effect of MMP on exercise performance

**Figure 3:**
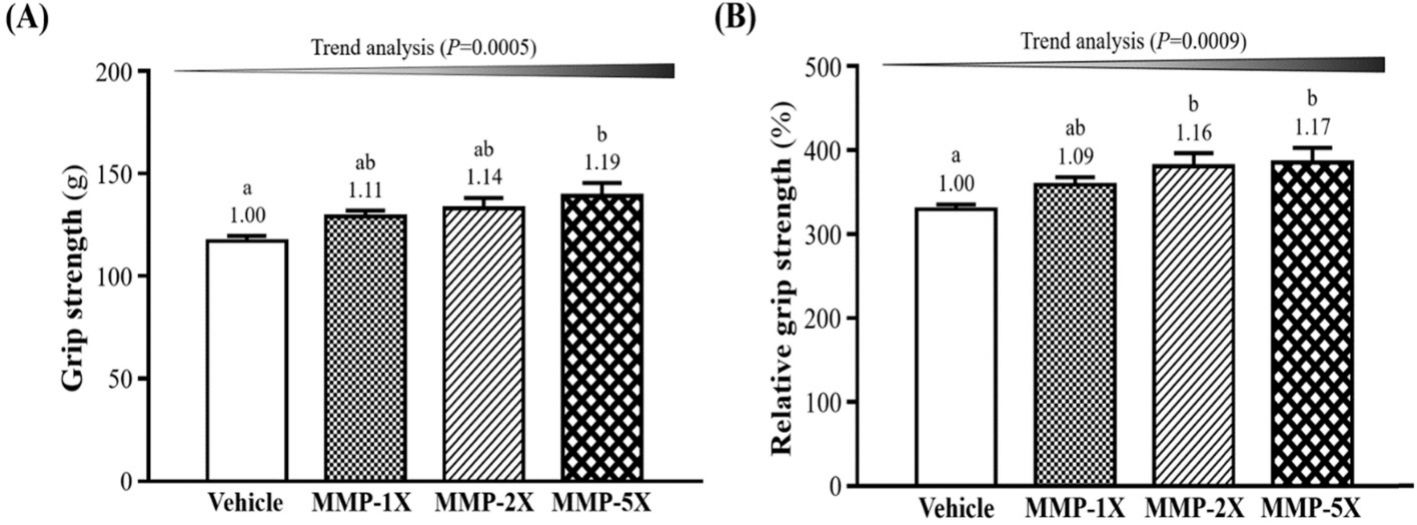
Effect of MMP supplementation on (A) forelimb grip strength; (B) relative grip strength (%). Data are presented as means±SEM (n =10). Bars with different superscript letters (a, b) are significantly different at *p* <0.05.

The two exercise performance tests contain grip strength test and exhaustive swimming exercise. As shown in **Figure 3A**, the mean grip strength in the vehicle, MMP-1X, MMP-2X and MMP-5X groups were 117±2.6, 129±2.9, 133±5.1 and 139±6.4 g, respectively. Compare with Vehicle and MMP-5X groups were significantly higher by 1.19-fold (*p* =0.007). The results of relative grip strength as calculate by normalizing dividing body weight of mice individually, was also significantly higher with MMP-2X (*p =*0.039) and MMP-5X (*p=*0.022) (**Figure 3B**). The effect of MMP has a significant dose effect on improving grip strength and relative grip strength (trend analysis,* p* <0.0005; *p* <0.0009).

**Figure 4:**
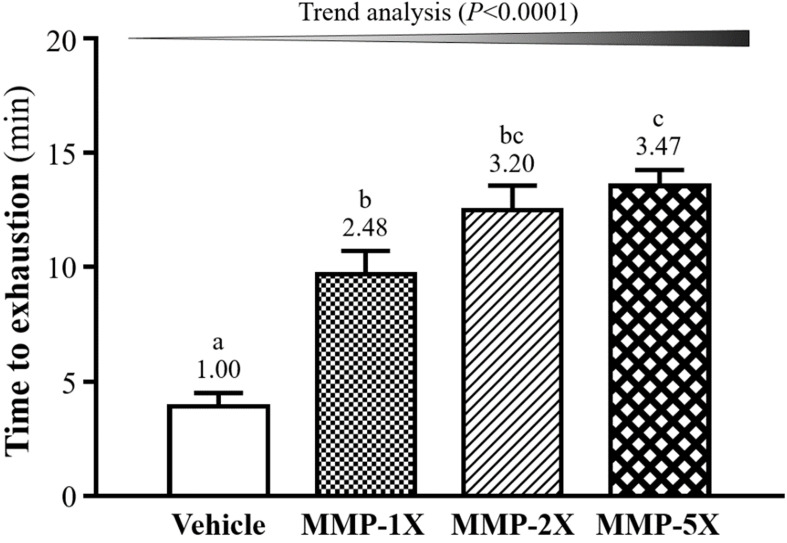
Effect of MMP on exhaustive swimming time. Data are presented as means±SEM (n =10). Bars with different superscript letters (a, b, c) are significantly different at *p* <0.05.

One of the physical performance tests including exhaustive swimming exercise. As seen in **Figure 4**, the endurance swimming time in the Vehicle, MMP-1X, MMP-2X and MMP-5X groups were 3.91±0.60, 9.71±1.00, 12.05±1.08 and 13.56±0.69 minutes, respectively. The swimming time of MMP-1X, MMP-2X and MMP-5X groups were significantly greater than the vehicle group by 2.48-fold, 3.20-fold and 3.47-fold (all *p* <0.0001). The effect of MMP has a significant dose effect on improving endurance swimming time (Trend analysis, *p* <0.0001).

### Effects of MMP supplementation on lactate post a 10-minute swimming test

Lactate levels were conducted pre-exercise, immediately after 10 min swimming test and 20 minutes after the rest (**Table 3**). Before swimming, there were no significant differences in the levels of blood lactate among each group. After 10 minutes swimming, serum lactate concentrations were significantly lower by 11.5% (*p* =0.004), 15.1% (*p* <0.0001) and 18.2% (*p* <0.0001) in the MMP-1X, MMP-2X and MMP-5X groups than the vehicle group. Levels of serum lactate after 20 minutes of rest were also significantly lower by 11.7%, 15.6% and 26.0% in the MMP-1X, MMP-2X and MMP-5X groups (all *p* <0.0001) than the vehicle group. The effects of MMP on lactate concentrations immediately after a 10-minute swimming and after a 20-minute rest were both dose-dependent (all *p* <0.0001).

The lactate ratios before and after the swimming test demonstrated that the accumulation of lactate was significantly decrease in the MMP-1X, MMP-2X and MMP-5X groups than in the vehicle group, by 12.7% (*p* =0.031), 15.1% (*p* =0.008), and 17.8% (*p* =0.001), respectively. Lactate clearance rates were 1.62-fold (*p* <0.0001) higher in the MMP-5X group than in the vehicle group. However, there was no significant difference between the vehicle, MMP-1X, MMP-2X groups.

### Effects of MMP supplementation on BUN and CK after a 90-minute swimming test and a 60-minute rest period

**Figure 5:**
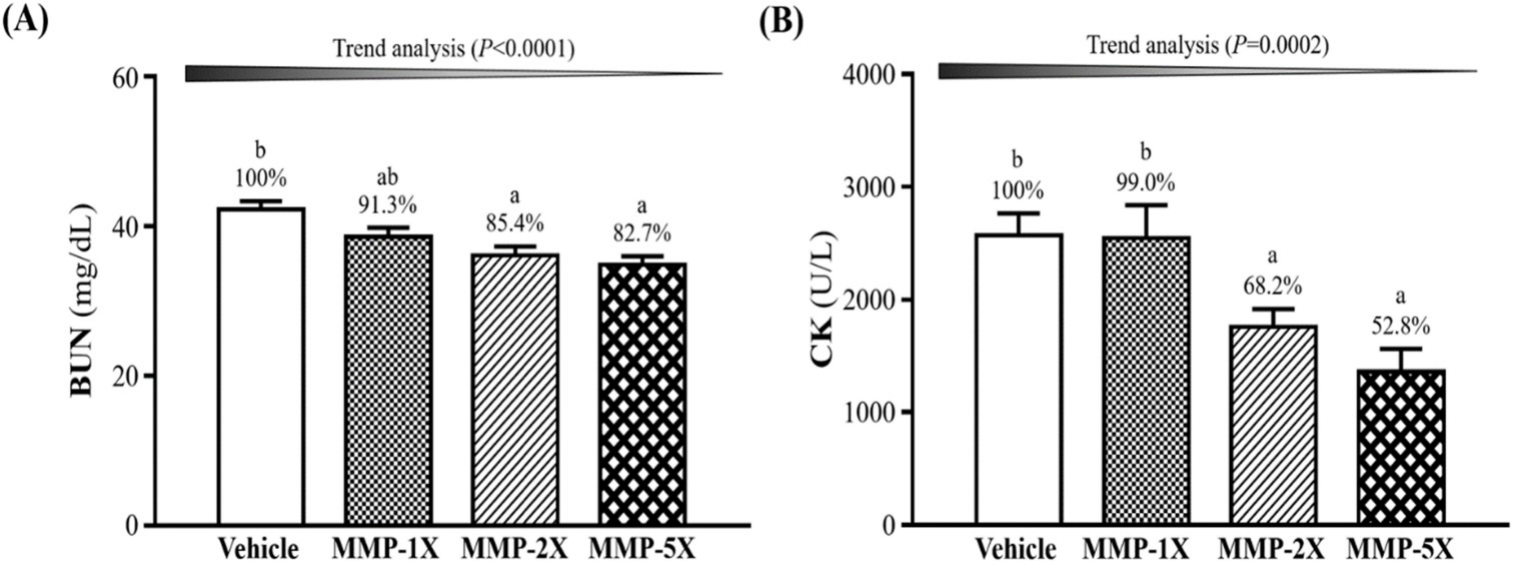
Effect of MMP on exhaustive swimming time. Data are presented as means±SEM (n =10). Bars with different superscript letters (a, b, c) are significantly different at *p* <0.05.

The BUN and CK levels were conducted immediately after 90 minutes swimming test and 60 minutes after the rest. As shown in **Figure 5A**, the BUN level after 90 minutes swimming and 60 minutes after the rest were significantly lower by 14.7% (*p* =0.004) and 17.5% (*p* =0.0001) in the MMP-2X and MMP-5X groups than the vehicle group. CK level with MMP-5X group was significantly lower by 47.2% (*p* =0.002) than the vehicle group (**Figure 5B**). The effect of MMP has a significant dose effect on decreasing BUN level (Trend analysis, *p* <0.0001).

### Effects of MMP supplementation on biochemical assessments

The results of biochemical at the end of the study could provide clinical information about the test animals' health status. The biochemical indices contain liver damage markers, renal, lipid-related metabolic and glucose metabolism biomarkers were also found to be unchanged between the groups (*p* >0.05, **Table 4**). The results of showed the doses of MMP supplementation used in the present study are safe with no effect on biochemical indices.

### Effect of MMP supplementation on liver and muscle glycogen determination

**Figure 6:**
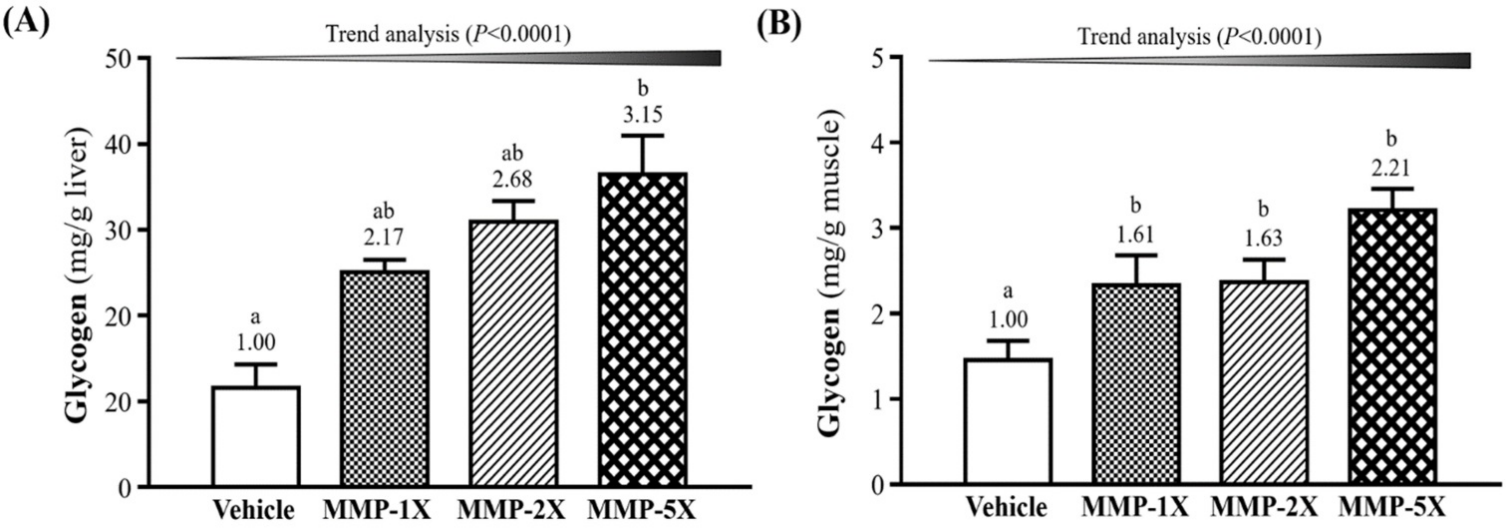
Effect of MMP on (A) hepatic glycogen and (B) muscle glycogen levels. Data are mean±SEM for n =10 mice per group. Bars with different superscript letters (a, b) are significantly different at *p* <0.05.

Glycogen is primarily content in the liver and skeletal muscles tissues for energy demands and homeostasis. The concentration of liver glycogen in vehicle, MMP-1X, MMP-2X and MMP-5X groups were 11.56±2.74, 25.05±1.49, 30.96±2.41, 36.43±4.56 mg/g, respectively (**Figure 6A**). The vehicle group was significantly lower than MMP-5X group by 3.15-fold (*p* <0.0001). The muscle glycogen, as seen in **Figure 6B**, vehicle, MMP-1X, MMP-2X and MMP-5X groups were 1.45±0.23, 2.33±0.35, 2.37±0.26, 3.21±0.25 mg/g, respectively.

Muscle glycogen levels of MMP-1X, MMP-2X and MMP-5X groups were significantly higher by 1.61- (*p* =0.016), 1.63- (*p* <0.0001) and 2.21-fold (*p* <0.0001) respectively, as compared with the vehicle group.

### Effects of MMP supplementation on histological observation

**Figure 7:**
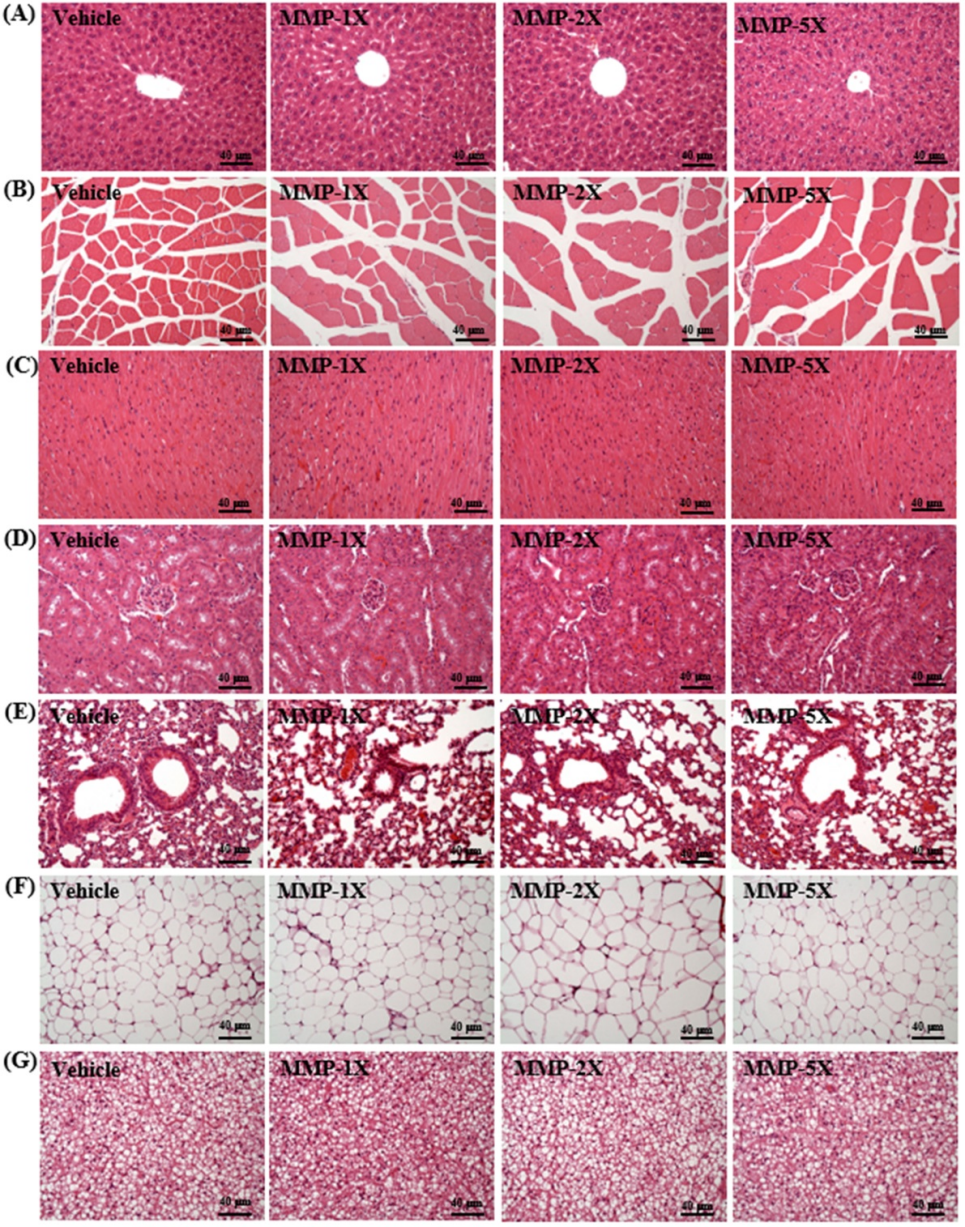
Effect of MMP on histomorphologic features of the (A) liver, (B) muscle, (C) heart, (D) kidney, (E) lung, (F) epididymal fay pads and (G) brown adipose tissue. Specimens were photographed under a light microscope. (H&E stain, magnification: 200×; bar, 40 µm).

**Figure 6** shows the histological staining - the four groups did not differ in the histological observations of the liver, muscle, heart, kidney, lung, EFP and BAT. Haematoxylin-eosin (H&E) staining showed that no significantly in liver tissues revealed normal hepatic architecture of hepatocytes, bile duct and sinusoid (**Figure 7A**). Hypertrophy and hyperplasia were not observed in muscle (**Figure 7B**) or heart (**Figure 7C**). The structures of renal tubules and the glomerulus did not differ between the groups (**Figure 7D**). All animals showed typical tissue architectures of the lung alveoli (**Figure 7E**). The adipose tissue or fat cell size was no difference in the morphology among all groups (**Figure 7F**). In BAT there are no difference in multiple lipid droplets among all groups (**Figure 7G**).

## Discussion

In this study, we investigated the effect of MMP supplementation on exercise performance and physical fatigue using a mouse model. Our study was conducted over a 14-days period which included grip strength test, exhaustive swimming test, fatigue-related biomarker measurement, and tissue glycogen storage capacity evaluation.

Our data showed that MMP supplementation could improve grip strength and relative grip strength, among them the MMP-5X group had the greatest forelimb grip strength than all group (Figure 2). However, the grip strength performance varies greatly depending on the bodyweight of mice 26. Since the grip strength is correlated to the body weight, the relative grip strength is calculated by forelimb grip strength divided by body weight to compare the performance between the group. The effect of MMP has a significant dose effect on improving grip strength and relative grip strength. Proteins present in mare's milk comprise 50-55% of slowly digested casein and 45% of rapidly digested globulins and albumins, combination of rapidly, slowly digested proteins, and carbohydrates (lactose) work together to improve muscle recovery and future performance 27, 28. In this study, MMP is rich in protein and lactose similar to those of cow's milk, may have contributed to the improvement of grip strength performance.

Swimming is an innate ability of mice, thus we selected swimming to evaluate the physical fitness performance 29. In addition, a weight-bearing swimming test was conducted since the higher intensity exercise elicited a higher level of fatigue response in a short time. We observed both the MMP groups had a longer swimming time when compared with the vehicle group. Hence, the results indicated that the MMP supplementation could benefit the exhaustive swimming performance. The spared glycogen in muscle and liver can lengthen the swimming time, thus enhanced the exhaustive swimming performance. The MMP is reported that contains L. helveticus, L. casei and L. plantarum 30. Our previous study has reported that the probiotic lactobacilli have a beneficial effect on exercise performance through lactate generation 31. Previous study demonstrated that the probiotic lactobacilli could supply the energy to host via lactate utilization after the acute exercise 32. As a result, the MMP supplementation may optimize the energy utilization by changing the gut microbiota diversity.

The results showed that powdered mare milk products are rich in high lactose (**Table 2**). The lactase in the intestine can break down the lactose into glucose and galactose to produce adenosine triphosphate as energy (ATP). The exhaustive swimming test was conducted in fasting, 30 minutes after the administration of whether water or MMP. There is one report showing that the pre-exercise nutrition rich in carbohydrate improved aerobic exercise performance in about 54% of literature, where the remaining literature reported the fasting did not have a significant effect on aerobic exercise 33. Another study about pre-exercise carbohydrate supplementation has shown that both glycogen level and aerobic exercise performance enhanced whether increased dietary intake of carbohydrate in the days pre-exercise or ingestion before exercise 3-4 hour 34. In this study, the improvement in exhaustive swimming time may be attributed to lactose in MMP.

It is reported that blood lactate levels increased after the acute exercise 35. When the lactate production rate overwhelms the clearance rate, blood lactate level rises. Concomitant with elevated lactate level, the accumulation of hydrogen ion reduces the phosphocreatine resynthesis rate, causing muscle acidification and thus inhibits glycolysis and generation of ATP 36, 37. There was no significant difference on baseline of blood lactate level between all groups (**Table 4**). Nevertheless, the MMP groups had significant lower levels of blood lactate when compared with the vehicle in the after 10 minutes swimming test and 20 minutes after the rest. The result of trend analysis indicated there was dose effect on the MMP supplementation, which represented the there is a greater improvement in post-exercise blood lactate level and rest blood lactate level with the increased amount of MMP supplementation. Interestingly, mares' milk had a high microbiological quality, which makes this product a valuable potential component of functional foods 38. Consistent with other study, immune-modulating properties of horse milk, has several beneficial effects on composition of gut microbiota, favoring the growth of Bifidobacterium spp. in the intestinal milieu after horse-milk ingestion 39. Our previous study found that the gut microbiota status has a beneficial effect on energy utilization, crucial for exercise performance 31, 40. Another factor that has contributed to lactate level improvement is the lactose content in MMP, which is required for growth of lactobacilli 41. Adequate lactose intake is reported that lactose has a beneficial effect on lactobacilli growth in the gut 42. As a result, MMP supplementation may improve gut microflora to increase blood lactate clearance in exercise and delay response of fatigue.

Blood urea nitrogen (BUN) is produced via the urea cycle in the liver, which is a waste product of catabolism of protein. Urea nitrogen is the main end product of protein catabolism and protein metabolism, elevations can be due to exercise type, is an major marker correlated with dehydration, protein breakdown, fatigue, and stress 36. We observed an increase in BUN in the athletes after the long-distance running, indicating the BUN level varies with intensity and duration of exercise 37. Concomitant with the increased intensity of exercise, there is an increase in blood ammonia level. Previous research noted that elevated blood ammonia leads to disturb neuropsychological function, which deleteriously alter exercise performance and induce fatigue 43. The result of BUN in our study is similar to previous study, the BUN level of mice increased after exercise. We observed that the MMP groups had lower BUN levels when compared with vehicle group, which the level decreased with the amount of MMP supplementation. Therefore, the MMP supplementation can reduce the protein breakdown rate and delay fatigue.

Creatine kinase (CK) is an enzyme exists in skeletal muscle. The serum CK concentration varies with pathological or physiological alteration 44. The elevated CK level can be found when tissue damage occurs in whether athletes or normal people 45, 46, so the serum CK level can be used for evaluating the muscle damage level. We found that the MMP-2X and MMP-5X group had significant lower CK levels when compared with vehicle group (Figure 4B). The result of the present study showed a dose effect on lowering post-exercise serum CK level of MMP supplementation. Consequently, the MMP supplementation can reduce the level of muscle damage.

Glycogen is a polymer that storage in the liver and muscle tissue which is composed of glucose. The glycogen in the liver plays an important role in glucose maintenance while the muscle glycogen serves energy to muscle cells through ATP generation 47, 48. When the glycogen content decreases, the function of muscle impaired in despite of alternative energy sources are available 49. In this study, the MMP powder is rich in high lactose. Lactose contains the simple sugar galactose and glucose, as the foundation for macromolecules, passes completely into hepatic absorption, and glycogen in the liver 50, 51. Galactose is metabolized in the liver where epimerization to glucose occurs while attached to Uridine diphosphate glucose (UDPG), yielding UDP-glucose, and pyrophosphorylase to glucose-1-phosphate is generated, the starting point for glycogen synthesis 52. Therefore, galactose absorbed from the gastrointestinal tract is converted to hepatic glycogen. In this study, MMP supplementation may promote of glycogen concentration in muscle and liver were elevated in MMP-supplemented groups. Therefore, the tissue glycogen storage content is a favorable indicator of fatigue. We found that the MMP groups had greater levels of liver and muscle glycogen stores when compared with the vehicle group (**Figure 5**). We observed the dose-effect on both liver and muscle glycogen storage capacity improvement of MMP supplementation. Consequently, the MMP supplementation can delay the response of fatigue via enhancement of liver and muscle glycogen storage capacity.

Mongolian mare's milk may be used as a supplement to meet most of our basic nutritional and health requirements. In pathological sections, we also did not detect any gross abnormalities or obvious lesions in the various tissues and organs. Taken together, we believe MMP treatment could be as a safe ergogenic aid for anti-fatigue and improving endurance performance.

## Conclusions

In conclusion, our results suggest that a 14-day supplementation with MMP could increase the grip strength and exhaustive swimming time in ICR mice. Besides, the administration of MMP has beneficial effects on post-exercise levels of blood lactate, BUN and CK, as well as the blood lactate clearance rate. Moreover, the MMP supplementation could significantly increase the hepatic and muscular glycogen levels. Finally, there is no health risk or tissue damage after the intervention of MMP. In conclusion, the MMP is a safe supplement of anti-fatigue with benefits including exercise performance improvement, increased energy storage capacity, and enhanced clearance rate of fatigue-related biomarker.

## Figures and Tables

**Table 1:** Nutrient composition of MMP

Nutrients	Content/100g MMP	%
Protein	17.7 g	19.51
Fat	1.2 g	2.98
Total amount of saturated fatty acids	0.4 g	-
Total amount of trans fatty acids	-	-
Carbohydrate	70.3 g	77.51
Sodium	187.1 mg	-
lactose	68.4 g	-
Total calories	362.8 kcal	-
Alanine	3.39	4.95
Arginine	1.58	2.31
Aspartic acid	6.78	9.90
Cysteine	1.54	2.25
Glutamic acid	13.28	19.39
Glycine	1.13	1.65
Histidine*	1.31	1.91
Isoleucine*^#^	4.16	6.07
Leucine*^#^	6.42	9.37
Lysine*	6.14	8.96
Methionine*	1.56	2.28
Phenylalanine*	2.16	3.15
Proline	4.53	6.61
Serine	3.05	4.45
Threonine*	4.45	6.50
Tryptophan*	1.13	1.65
Tyrosine	1.79	2.61
Valine*^#^	4.10	5.99
Total AA	68.5	100
Total EAA*	31.43	45.88
Total BCAA^#^	14.68	21.52

Essential amino acids (EAA) are denoted by *. Branched chain acids (BCAA) are denoted by #.

**Table 2:** General characteristics of the experimental groups

Characteristic	Vehicle	MMP-1X	MMP-2X	MMP-5X	Trend Analysis
Initial BW (g)	31.4±0.2	31.6±0.2	31.6±0.2	31.5±0.2	0.444
Final BW (g)	36.0±0.6	36.0±0.3	36.0±0.6	36.4±0.7	0.7008
Food intake (g/day)	7.5±0.1	7.4±0.2	7.5±0.2	7.3±0.2	0.4134
Water intake (mL/day)	7.3±0.1	7.3±0.1	7.3±0.0	7.3±0.0	0.2073
**Weight (g)**					
Liver	2.13±0.06	2.13±0.05	2.13±0.08	2.16±0.04	0.5758
Muscle	0.35±0.01	0.36±0.00	0.36±0.01	0.37±0.01	0.1179
Kidney	0.64±0.02	0.63±0.02	0.63±0.02	0.64±0.02	0.7926
Heart	0.19±0.01	0.19±0.01	0.19±0.01	0.19±0.01	0.8393
Lung	0.23±0.01	0.23±0.01	0.23±0.01	0.23±0.01	0.9337
EFP	0.16±0.01^b^	0.12±0.01^a^	0.12±0.01^a^	0.12±0.01^a^	0.1101
BAT	0.28±0.02	0.35±0.02	0.34±0.3	0.36±0.03	0.0274
**Relative Weight (%)**					
liver	5.91±0.11	5.91±0.12	5.90±0.14	5.93±0.07	0.5966
Muscle	0.97±0.03	0.99±0.02	0.99±0.02	1.01±0.02	0.0658
Kidney	1.78±0.05	1.74±0.06	1.75±0.05	1.75±0.02	0.5467
Heart	0.53±0.03	0.53±0.03	0.52±0.02	0.51±0.02	0.3153
Lung	0.65±0.04	0.65±0.04	0.63±0.02	0.63±0.03	0.6355
EFP	0.44±0.02^b^	0.33±0.03^a^	0.34±0.02^a^	0.32±0.03^a^	0.0026
BAT	0.78±0.06	0.98±0.06	0.94±0.08	0.98±0.06	0.0524

Data are presented as means ± SEM (n =10). Data were analysed by one-way ANOVA, values in the same row with different superscript letters (a, b) differed significantly, *p* <0.05. EFP: epididymal fat pad; BAT: brown adipose tissue.

**Table 3:** Effects of MMP supplementation on lactate levels during acute exercise challenge

Time Point	Vehicle	MMP-1X	MMP-2X	MMP-5X	Trend Analysis
	Lactate (mmol/L)				
Before swimming (A)	3.15±0.11	3.17±0.05	3.14±0.06	3.14±0.09	0.6724
After swimming (B)	8.80±0.19^b^	7.79±0.14^a^	7.47±0.20^a^	7.20±0.23^a^	<0.0001
After a 20-minute rest (C)	7.7±0.17^c^	6.8±0.10^b^	6.5±0.14^b^	5.7±0.17^a^	<0.0001
	**Lactate production rate and clearance rate**				
Production rate = B/A	2.82±0.10^b^	2.46±0.06^a^	2.39±0.09^a^	2.31±0.10^a^	<0.0001
Clearance rate = (B-C)/B	0.12±0.00^a^	0.13±0.01^a^	0.13±0.01^a^	0.20±0.01^b^	0.0004

The metabolite lactate was assessed for the four groups: vehicle, MMP-1X, MMP-2X, and MMP-5X at three points. Production rate of lactate (B/A) was calculated as the lactate level after exercise (B) divided by the lactate level before exercise (A). Clearance rate of lactate was difference between after exercise and after rest divided by after rest. Data are presented as means ± SEM (n =10).Values in the same row with different superscript letters (a, b) differed significantly, *p* <0.05.

**Table 4:** Effects of MMP supplementation on biochemical indices at the end of the experiment

Characteristics	Vehicle	MMP-1X	MMP-2X	MMP-5X	Trend Analysis
AST (U/L)	70±4	69±4	69±3	67±4	0.6644
ALT (U/L)	44±2	43±4	42±4	43±4	0.4767
Albumin (g/dL)	2.9±0.0	3.0±0.0	3.0±0.0	3.0±0.1	0.3609
TP (g/dL)	5.1±0.0	5.2±0.1	5.2±0.0	5.2±0.1	0.2383
BUN (mg/dL)	23.9±0.5	23.3±0.4	23.7±0.8	23.9±0.7	0.7909
Creatinine (g/dL)	0.40±0.01	0.41±0.01	0.40±0.01	0.41±0.01	0.3721
UA (mg/dL)	2.9±0.2	2.9±0.3	2.9±0.3	2.9±0.1	0.7739
CK (U/L)	138±16	106±20	101±9	104±14	0.2571
TC (mg/dL)	143±3	144±4	143±6	145±5	0.8935
TG (mg/dL)	157±6	163±15	162±16	163±6	0.4321
Glucose (mg/dL)	192±8	193±5	195± 3	194±5	0.9759

Data are presented as means±SEM (n =10). Values in the same row with different superscript letters (a, b) differed significantly, *p* <0.05. AST, aspartate aminotransferase; ALT, alanine aminotransferase; TP, total protein; BUN, blood urea nitrogen; UA, uric acid; CK, creatine kinase; TC, total cholesterol; TG, triacylglycerol.
